# Supplementary material for: An at-leg pellet and associated Penicillium sp. provide multiple protections to mealybugs
Source: Commun Biol. 2024 May 16;7:580. doi: 10.1038/s42003-024-06287-2 (PMC11099121; doi:10.1038/s42003-024-06287-2)
Supplement: Supplementary file 2 — Supplementary Information [file 42003_2024_6287_MOESM2_ESM.pdf]

Supplementary Material

An at-leg pellet and associated *Penicillium* sp. provide multiple protections to mealybugs

Zicheng Li[1,†], Haojie Tong[1,2,†], Meihong Ni[1], Yiran Zheng[1], Xinyi Yang[1], Yumei Tan[1], Zihao Li[1], Mingxing Jiang[1*]

[1]Institute of Insect Sciences, Key Laboratory of Biology of Crop Pathogens and Insects of Zhejiang Province, Key Laboratory of Molecular Biology of Crop Pathogens and Insects, Ministry of Agriculture, State Key Laboratory of Rice Biology, Zhejiang University, Hangzhou 310058, China.

[2]College of Life Sciences, China Jiliang University, Hangzhou 310018, China.

This PDF includes:

Supplementary Figures:

Supplementary Figure. 1 Formation status of pellets in different Pseudococcidae insects on different hosts.

Supplementary Figure. 2 Maximum likelihood tree illustrating the phylogeny of *Penicillium citrinum* and other *Penicillium* spp. based on ITS nuclear rDNA sequences dataset.

Supplementary Figure. 3 Morphology of *P. citrinum* from field-collected mealybug's legs on different medium.

Supplementary Figure. 4 Antimicrobial ability assays of rice extract against *Akantaomyces lecanii* (a), *Beauveria bassiana* (b), *Pseudomonas syringae* (c) and *Botrytis cinerea* (d) using the disk-diffusion method.

Supplementary Figure. 5 Fungi isolated from field-collected *P. solenopsis* after removing the legs.

Supplementary Figure. 6 Concentration of 2,4-DTBP in pellets of mealybug (a) and its inhibition activity again *A. lecanii* (b) and *B. bassiana* (c) at different concentrations.

Supplementary Figure. 7 Fungi isolated from dead *P. solenopsis* after being sprayed with *A. lecanii* spores and incubated for 24 h (a) and 72 h (b).

Supplementary Figure. 8 Effects of cotton mealybug crawling on tomato leaves.

Supplementary Figure. 9 Sucking waveform of *P. solenopsis* without at-leg pellets (a) and with pellets (b).

Supplementary Tables:

Supplementary Table 1. Top ten compounds in the fermentation product of *P.citrinum*

Supplementary Table 2. Reference sequences (ITS) used in phylogenetic analyses

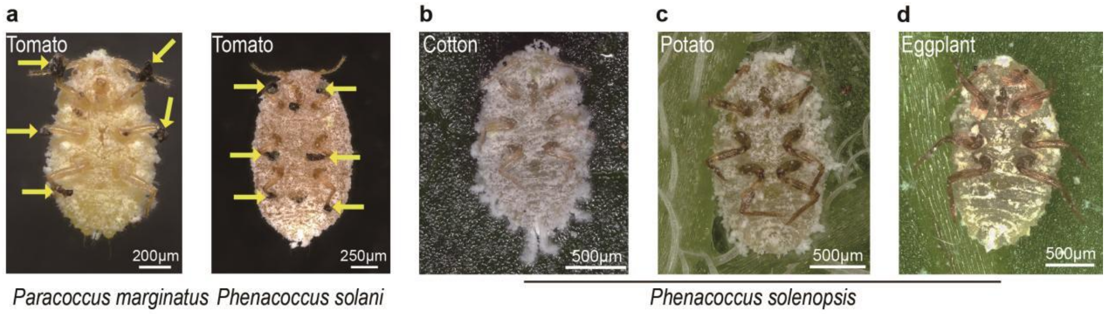

Supplementary Figure. 1 Formation status of pellets in different Pseudococcidae insects on different hosts. (a) Pellets (yellow arrows) formed at the legs of adult *Paracoccus marginatus* female (left) and *Phenacoccus solani* female (right) 24 h after molting and feeding on tomato plants. No pellets can be formed on adult *Phenacoccus solenopsis* female feeding on cotton (b), potato (d), and eggplant (d).

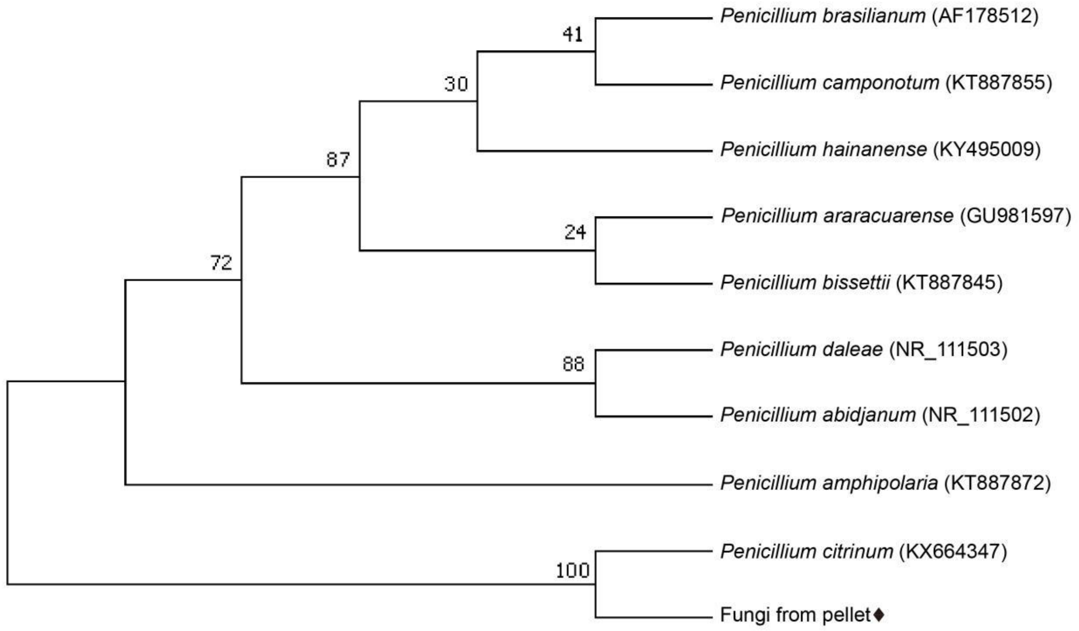

Supplementary Figure. 2 Maximum likelihood tree illustrating the phylogeny of *Penicillium citrinum* and other *Penicillium* spp. based on ITS nuclear rDNA sequences dataset. The diamond indicates the isolate from this study, while all other accessions are collected reference sequences. ML ultrafast bootstrap supports are shown above branches.

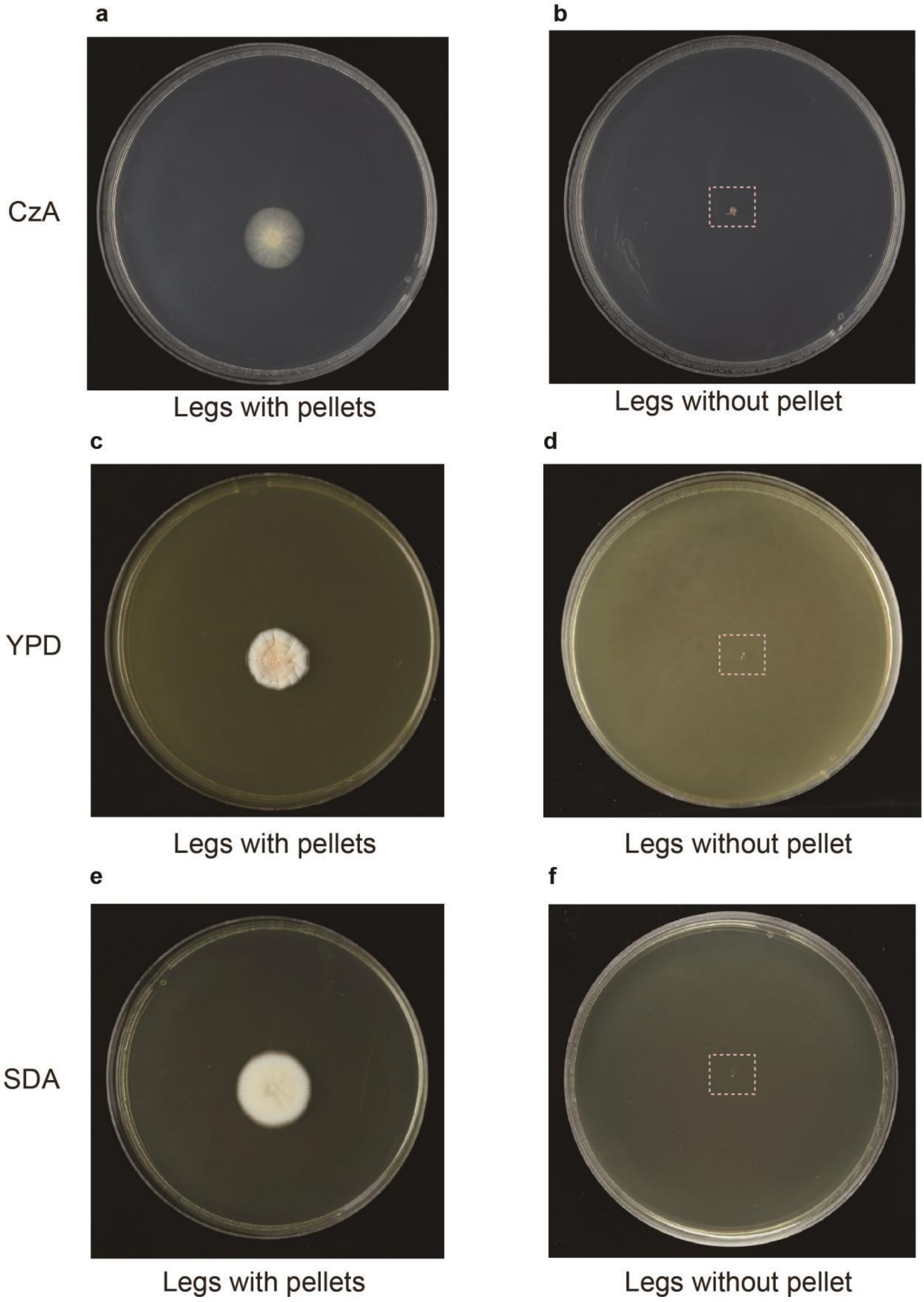

Supplementary Figure. 3 Morphology of *P. citrinum* from field-collected mealybug's legs on different medium. *P. citrinum* colony was generated from mealybug legs with pellets on Czapek dox agar (CzA) medium (a), Yeast extract peptone dextrose (YPD) medium (c), and Sabouraud dextrose agar (SDA) medium (e). No fungi were isolated from legs of mealybugs without pellets on CzA (b), YPD (d), and SDA (f).

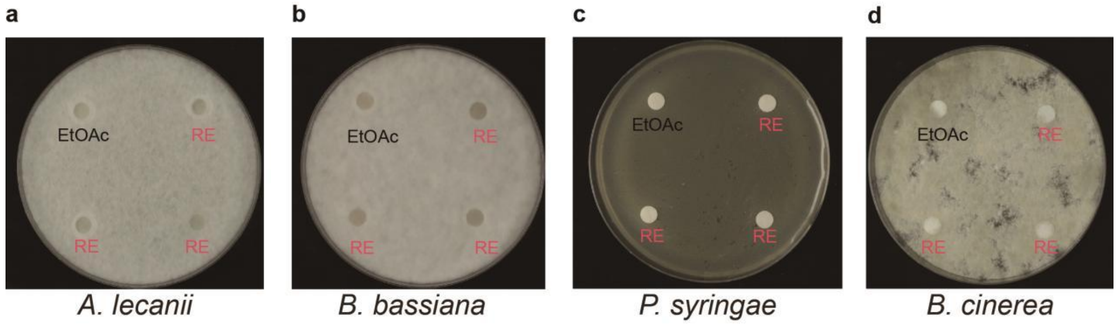

Supplementary Figure. 4 Antimicrobial ability assays of rice extract against *Akantaomyces lecanii* (a), *Beauveria bassiana* (b), *Pseudomonas syringae* (c) and *Botrytis cinerea* (d) using the disk-diffusion method. EtOAc: Ethyl acetate; RE: rice extract containing PDA liquid medium.

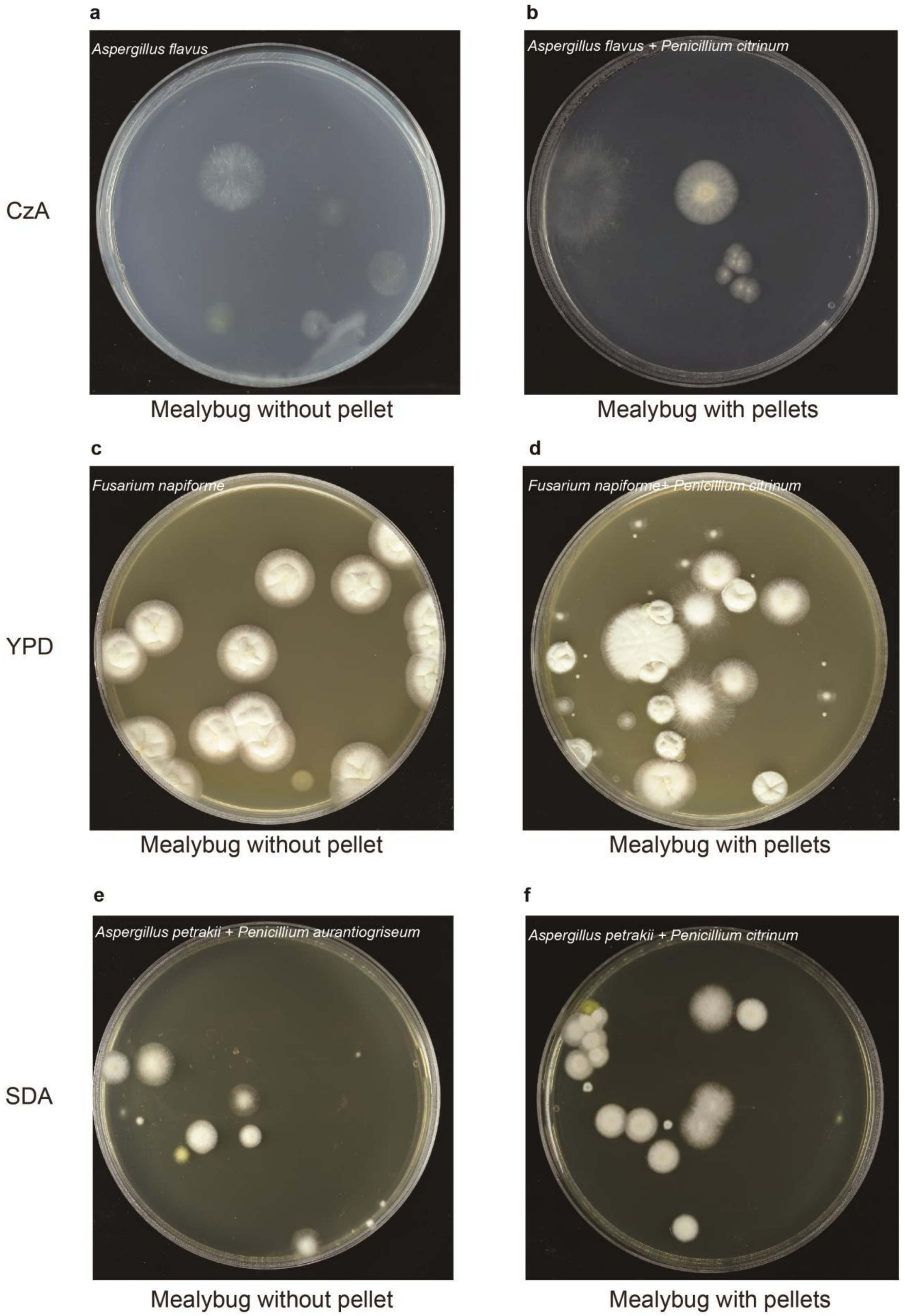

Supplementary Figure. 5 Fungi isolated from field-collected *P. solenopsis* after removing the legs. *P. solenopsis* without pellets and those with pellets at collection were showed on the left and right, respectively. Medium of CzA (ab), YPD (cd), and SDA (ef) were used. The fungi isolated were labeled in the upper left.

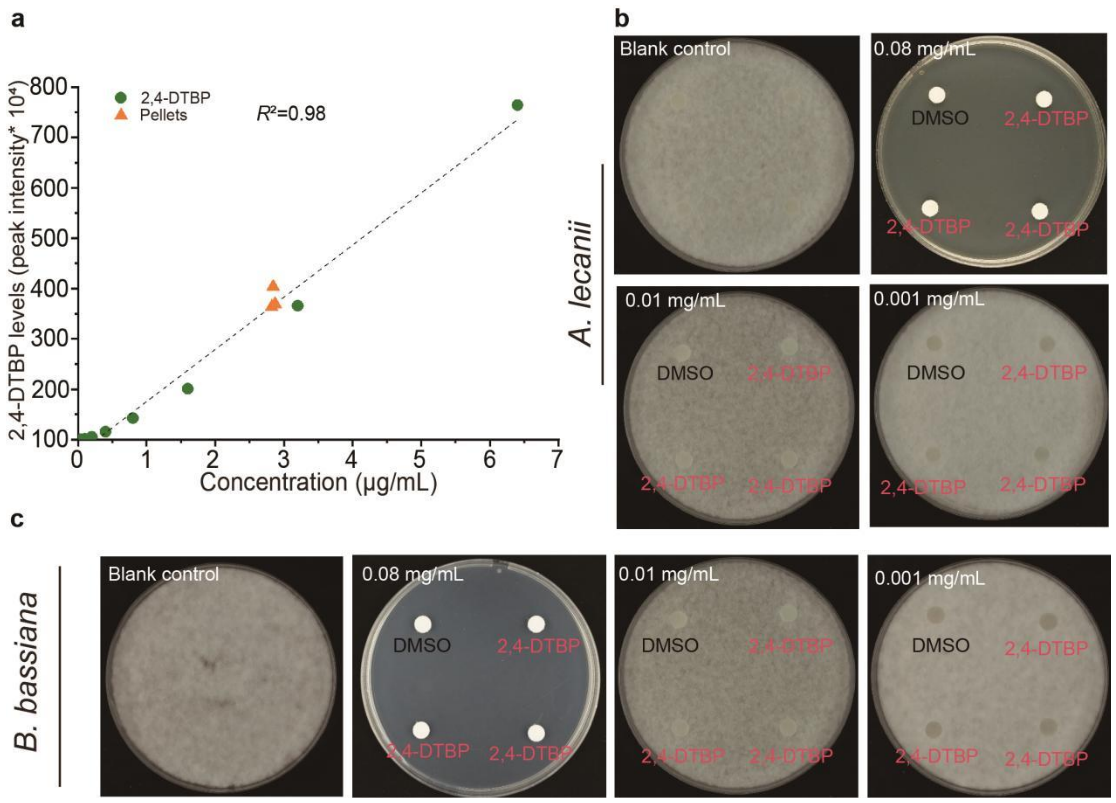

Supplementary Figure. 6 Concentration of 2,4-DTBP in pellets of mealybug (a) and its inhibition activity again *A. lecanii* (b) and *B. bassiana* (c) at different concentrations. Concentrations of 2,4-DTBP were showed on top left of each photograph. The first microbe-containing plate is a blank control.

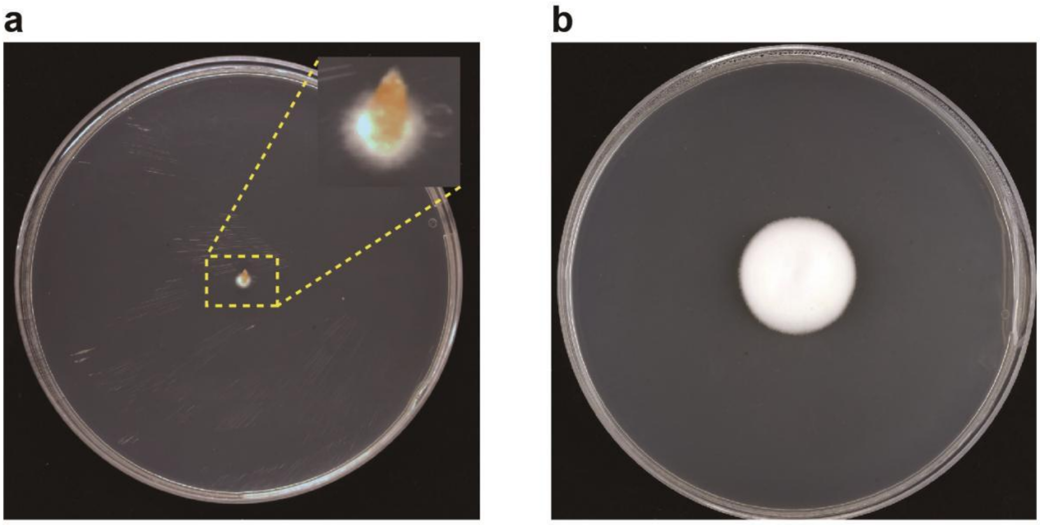

Supplementary Figure. 7 Fungi isolated from dead *P. solenopsis* after being sprayed with *A. lecanii* spores and incubated for 24 h (a) and 72 h (b).

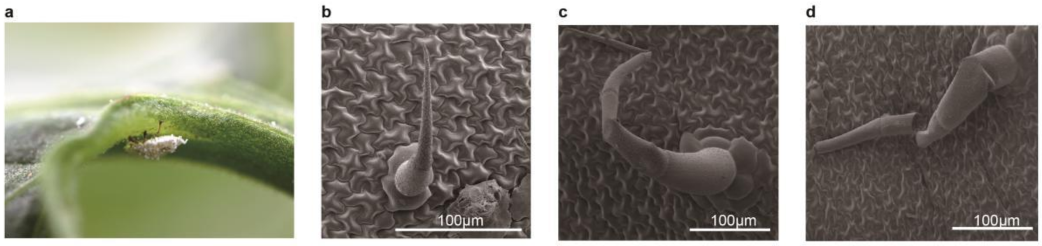

Supplementary Figure. 8 Effects of cotton mealybug crawling on tomato leaves. (a) Crawling posture of mealybugs. Glandular trichome on the leaves free of mealybugs (b) and on the leaves experiencing crawling of mealybugs with pellets (c) or crawling of mealybugs without pellet (d) for 10 min, observed under SEM.

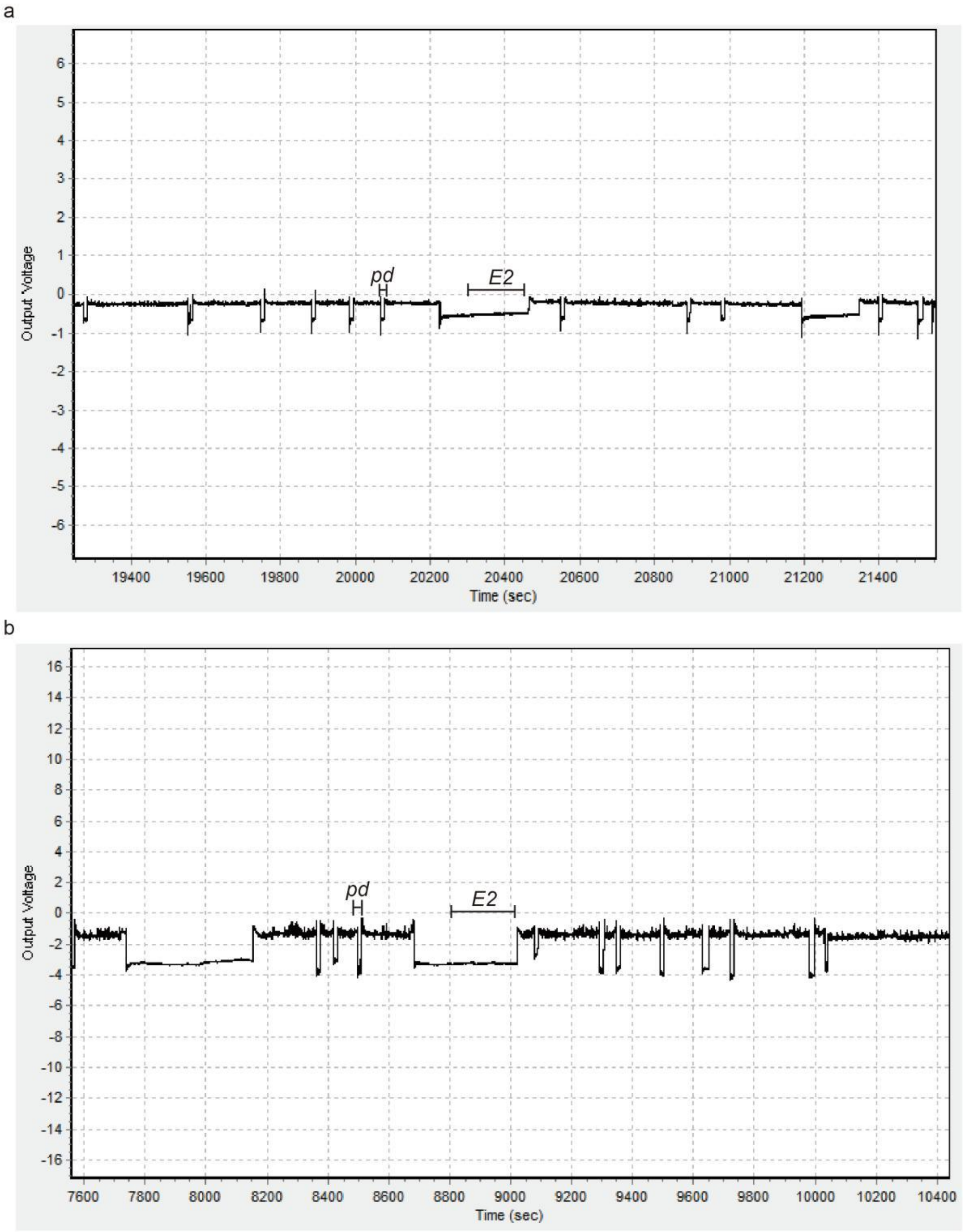

Supplementary Figure. 9 Sucking waveform of *P. solenopsis* without at-leg pellets (a) and with pellets (b). Waveforms *pd*: formed when the mouthparts of mealybugs probed mesophyll cells; waveforms *E2*: formed when the mouthparts probed the phloem**.**

Supplementary Table 1. Top ten compounds in the fermentation product of *P. citrinum*

Compound	Retention time (min)	Ion mode	Molecular weight	Compound	Retention time (min)	Ion mode	Molecular weight
(2E)-N-(4-acetamidobutyl)-3-(4-hydroxy-3-methoxyphenyl)prop-2-enamide	9.86	N	306.15	Citrinin	12.859	P	250.08
Butylparaben	7.469	N	194.09	4-(2,3-dihydro-1,4-benzodioxin-6-ylsulfonylamino)butanoic acid	10.375	P	222.09
5-hex-1-ynylfuran-2-carboxylic acid	9.512	N	192.08	3-methyl-2-oxobutanoic acid	7.353	P	116.05
Eugenol	10.792	N	164.08	Adenine	1.532	P	135.05
Ethyl b-d-glucuronide	2.152	N	222.07	N-acetyl-phenylalanin	9.502	P	207.09
4-Acetamidobutanoic acid	6.046	N	145.07	4-(4-methoxyphenyl)-6-pyridin-4-yl-1,3,5-triazin-2(3H)-one	11.438	P	280.09
N-acetyl-L-tyrosine	4.243	N	223.08	2-acetamido-3-(4-methoxyphenyl) propanoic acid	7.977	P	238. 11
2,6-dihydroxypurine	2.272	N	152.03	3-indoleacetic	10.644	P	175.06
N-acetyl-L-	7.723	N	207.09	Caprolactam	7.552	P	113.08
Inosine	2.158	N	268.08	Diethyleneglycol	8.502	P	190.08

Supplementary Table 2. Reference sequences (ITS) used in phylogenetic analyses

Species	GenBank numbers
Penicillium hainanense	KY495009
Penicillium brasilianum	AF178512
Penicillium camponotum	KT887855
Penicillium araracuarense	GU981597
Penicillium bissettii	KT887845
Penicillium daleae	NR_111503
Penicillium abidjanum	NR_111502
Penicillium amphipolaria	KT887872
Penicillium citrinum	KX664347
