# Supplementary material for: An at-leg pellet and associated Penicillium sp. provide multiple protections to mealybugs
Source: Commun Biol. 2024 May 16;7:580. doi: 10.1038/s42003-024-06287-2 (PMC11099121; doi:10.1038/s42003-024-06287-2)
Supplement: Supplementary file 5 — Reporting Summary [file 42003_2024_6287_MOESM5_ESM.pdf]

nature portfolio

Corresponding author(s): Mingxing Jiang

Last updated by author(s): Apr 7, 2024

Reporting Summary

Nature Portfolio wishes to improve the reproducibility of the work that we publish. This form provides structure for consistency and transparency in reporting. For further information on Nature Portfolio policies, see our Editorial Policies and the Editorial Policy Checklist.

Statistics

For all statistical analyses, confirm that the following items are present in the figure legend, table legend, main text, or Methods section.

n/a	Confirmed	
☐	☒	The exact sample size (*n*) for each experimental group/condition, given as a discrete number and unit of measurement
☐	☒	A statement on whether measurements were taken from distinct samples or whether the same sample was measured repeatedly
☐	☒	The statistical test(s) used AND whether they are one- or two-sided<br>*Only common tests should be described solely by name; describe more complex techniques in the Methods section.*
☒	☐	A description of all covariates tested
☐	☒	A description of any assumptions or corrections, such as tests of normality and adjustment for multiple comparisons
☐	☒	A full description of the statistical parameters including central tendency (e.g. means) or other basic estimates (e.g. regression coefficient) AND variation (e.g. standard deviation) or associated estimates of uncertainty (e.g. confidence intervals)
☐	☒	For null hypothesis testing, the test statistic (e.g. *F*, *t*, *r*) with confidence intervals, effect sizes, degrees of freedom and *P* value noted<br>*Give P values as exact values whenever suitable.*
☒	☐	For Bayesian analysis, information on the choice of priors and Markov chain Monte Carlo settings
☒	☐	For hierarchical and complex designs, identification of the appropriate level for tests and full reporting of outcomes
☒	☐	Estimates of effect sizes (e.g. Cohen's *d*, Pearson's *r*), indicating how they were calculated

Our web collection on statistics for biologists contains articles on many of the points above.

Software and code

Policy information about availability of computer code

Data collection	MEGA (7.0), Compound Discoverer (3.1), TRACE (1310), Chromeleon (7.2), SPSS (25.0), and GraphPad Prism (9.0)
Data analysis	MEGA (7.0), Compound Discoverer (3.1), TRACE (1310), Chromeleon (7.2), SPSS (25.0), and GraphPad Prism (9.0)

For manuscripts utilizing custom algorithms or software that are central to the research but not yet described in published literature, software must be made available to editors and reviewers. We strongly encourage code deposition in a community repository (e.g. GitHub). See the Nature Portfolio guidelines for submitting code & software for further information.

Data

Policy information about availability of data

All manuscripts must include a data availability statement. This statement should provide the following information, where applicable:

- Accession codes, unique identifiers, or web links for publicly available datasets
- A description of any restrictions on data availability
- For clinical datasets or third party data, please ensure that the statement adheres to our policy

All data are available in the manuscript or the supplementary data. The DNA sequencing data for ITS nuclear rDNA sequences generated from P. citrinum is available under the accession code of GenBank ID: OR647500. And the DNA sequencing data for ITS nuclear rDNA sequences generated from Aspergillus sp. and Cercosporais sp. is available under the accession code of GenBank ID: PP338193 and PP338195.

Research involving human participants, their data, or biological material

Policy information about studies with human participants or human data. See also policy information about sex, gender (identity/presentation), and sexual orientation and race, ethnicity and racism.

Reporting on sex and gender	Not applicable
Reporting on race, ethnicity, or other socially relevant groupings	Not applicable
Population characteristics	Not applicable
Recruitment	Not applicable
Ethics oversight	Not applicable

Note that full information on the approval of the study protocol must also be provided in the manuscript.

Field-specific reporting

Please select the one below that is the best fit for your research. If you are not sure, read the appropriate sections before making your selection.

☒ Life sciences ☐ Behavioural & social sciences ☐ Ecological, evolutionary & environmental sciences

For a reference copy of the document with all sections, see nature.com/documents/nr-reporting-summary-flat.pdf

Life sciences study design

All studies must disclose on these points even when the disclosure is negative.

Sample size	For the formation of pellets in a greenhouse, sample size n>30; formation of pellets on field-grown tomato, sample size n=30; the loss and reacquisition of pellets, sample size n=30; formation of pellets on different plants, sample size n=50; formation of pellets on different tomato leaves, sample size n=19-29 for intact tomato leaves and sample size n=23-30 for ultrasonic surface cleaned tomato leaves; formation of pellets on the different generation, sample size n=20-91 for the first generation and sample size n=48-147 for the second generation; colonization of Penicillium citrinum in honeydew, sample size n=3; the percentage of mealybugs carrying P. citrinum, sample size n=30 on tomato previously treated with microbe-free honeydew and sample size n=30-32 for the honeydew inoculated with P. citrinum; the primary components of pellets, sample size n=12000; the presence of 2,4-DTBP in tomato leaves and the fermentation product of P. citrinum, sample size n=3; the survival rate of mealybugs, sample size n=29-32 for the mealybugs with pellets and sample size n=18-26 without pellets; PIN2 gene expression in tomatoes, sample size n=5; EPG analysis, sample size n=29 for mealybugs with pellets and n=29 for mealybugs without pellets;the presence of butylparaben and citrinin in pellets (sample size n=12000); the concentration of 2,4-DTBP in pellets, sample size n=12000.
Data exclusions	All the data does not meet the acceptance criteria and all the outliers are excluded.
Replication	For the formation of pellets under different condition, replication n=5; primary components of the fermentation product of P. citrinum, pellets and tomato, replication n=1; the presence of butylparaben and citrinin in pellets, replication n=1; the competitive ability of P. citrinum, replication n=10; the survival rate of mealybugs exposed to Verticillium Lecanii, replication n=5; others, replication n=3.
Randomization	Animal study are randomized.
Blinding	Animal study are double-blinded.

Reporting for specific materials, systems and methods

We require information from authors about some types of materials, experimental systems and methods used in many studies. Here, indicate whether each material, system or method listed is relevant to your study. If you are not sure if a list item applies to your research, read the appropriate section before selecting a response.

Materials & experimental systems

n/a	Involved in the study
☒	☐ Antibodies
☒	☐ Eukaryotic cell lines
☒	☐ Palaeontology and archaeology
☐	☒ Animals and other organisms
☒	☐ Clinical data
☒	☐ Dual use research of concern
☐	☒ Plants

Methods

n/a	Involved in the study
☒	☐ ChIP-seq
☒	☐ Flow cytometry
☒	☐ MRI-based neuroimaging

Animals and other research organisms

Policy information about studies involving animals; ARRIVE guidelines recommended for reporting animal research, and Sex and Gender in Research

Laboratory animals	Phenacoccus solenopsis were collected from Hibiscus mutabilis plants in Hengfan, Lanxi, Zhejiang Province, China; Phenacoccus solani were collected from Lithops sp. in Yushi Valley, Hangzhou, Zhejiang Province, China; and Paracoccus marginatus were collected from papaya Carica papaya adjacent to Hainan University, Danzhou, Hainan Province, China. The collected insects were reared in screened cages (40 cm 50 cm 50 cm) at the Zijingang Campus of Zhejiang University (ZU) with potted tomato and cotton for 2-4 generations prior to use in a phytotron maintained at 26 ± 1°C and RH 70 ± 5% with a photoperiod of 14 h : 10 h (L : D). P. solenopsis were also reared in cages with potted tomato in a greenhouse (26 ± 5°C) and a field at the campus.
Wild animals	Not applicable.
Reporting on sex	Not applicable.
Field-collected samples	Phenacoccus solenopsis were collected from field-grown tomato on Zijingang Campus of Zhejiang University.
Ethics oversight	Not applicable.

Note that full information on the approval of the study protocol must also be provided in the manuscript.

Dual use research of concern

Policy information about dual use research of concern

Hazards

Could the accidental, deliberate or reckless misuse of agents or technologies generated in the work, or the application of information presented in the manuscript, pose a threat to:

No	Yes
☒	☐ Public health
☒	☐ National security
☒	☐ Crops and/or livestock
☒	☐ Ecosystems
☒	☐ Any other significant area

Experiments of concern

Does the work involve any of these experiments of concern:

No	Yes	
☒	☐	Demonstrate how to render a vaccine ineffective
☒	☐	Confer resistance to therapeutically useful antibiotics or antiviral agents
☒	☐	Enhance the virulence of a pathogen or render a nonpathogen virulent
☒	☐	Increase transmissibility of a pathogen
☒	☐	Alter the host range of a pathogen
☒	☐	Enable evasion of diagnostic/detection modalities
☒	☐	Enable the weaponization of a biological agent or toxin
☒	☐	Any other potentially harmful combination of experiments and agents

Plants

Seed stocks

1) Solanum lycopersicum: cv. Cooperative 903, Shanghai Changzhong Seeds Industry Co., Ltd, China;
2) Gossypium hirsutum: cv. Zhemian 1793, supplied by Prof. Shuijin Zhu from Zhejiang University;
3) Solanum tuberosum: cv. Netherlands Fifteen, Qingdao Jinmandi potato Co., Ltd, China; and
4) Solanum melongena: cv. Yinqie, Zhejiang Academy of Agricultural Sciences, China).

Novel plant genotypes

Not applicable

Authentication

Not applicable
